# Editorial: Development and validation of new molecular probes of nuclear medicine and new targets of nuclear drugs in cancers

**DOI:** 10.3389/fonc.2026.1812790

**Published:** 2026-03-16

**Authors:** Wanying Li, Ziyang Wang, Xiaoliang Chen, Yong Qin, Dong Dai

**Affiliations:** 1 School of Medical Technology, Tianjin University of Traditional Chinese Medicine, Tianjin, China; 2 Department of Nuclear Medicine, National Clinical Research Center for Cancer, Tianjin Cancer Hospital Airport Hospital, Tianjin, China; 3 Department of Nuclear Medicine, Chongqing University Cancer Hospital, Chongqing, China; 4 Department of Pharmaceutical Sciences, School of Pharmacy, The University of Texas at El Paso, El Paso, TX, United States; 5 Department of Molecular Imaging and Nuclear Medicine, Tianjin Medical University Cancer Institute and Hospital, National Clinical Research Center for Cancer, Tianjin, China

**Keywords:** imaging biomarkers, precision oncology, radiopharmaceuticals, targeted radionuclide therapy, theranostics

## Introduction

Nuclear medicine is undergoing a rapid transition from conventional diagnostic imaging to integrated theranostics, establishing itself as a key component of precision oncology. Advances in radiochemistry, molecular biology, and quantitative imaging have converged to create unprecedented opportunities for both tumor characterization and targeted treatment. This Research Topic compiles recent work that spans fundamental research on drug resistance mechanisms, radiopharmaceutical development, and clinical imaging applications, collectively illustrating the breadth and depth of progress in this evolving field.

## Tumor drug resistance: new mechanisms and targets

Overcoming drug resistance remains one of the most pressing challenges in oncology. Xu et al. systematically reviewed cuproptosis, a recently characterized form of regulated cell death driven by the disruption of intracellular copper homeostasis. Excess copper triggers the aggregation of lipoylated mitochondrial proteins and the loss of iron–sulfur cluster proteins within the tricarboxylic acid cycle, ultimately leading to proteotoxic stress and cell death. The authors further discussed how copper ionophores, copper chelators, and engineered nanodelivery systems may be harnessed to exploit this pathway against drug-resistant tumors. In a complementary study on hematologic malignancy, Li et al. investigated the role of sperm-associated antigen 6 (SPAG6) in multiple myeloma. Using bioinformatics analysis, clinical bone marrow specimens, and *in vitro* functional assays, they demonstrated that SPAG6 is markedly overexpressed in myeloma cells and correlates with elevated blood calcium, increased plasma cell ratio, and skeletal infiltration. Mechanistically, SPAG6 interacts with dual-specificity phosphatase 1 (DUSP1) to activate the MAPK/ERK signaling cascade, promoting cell proliferation, migration, and resistance to apoptosis. These results position SPAG6 as a promising therapeutic target for multiple myeloma.

## Radiopharmaceutical development and therapeutic innovation

The design of efficient molecular probes underpins the advancement of precision theranostics. Luo et al. provided a comprehensive review of fibroblast activation protein inhibitor (FAPI)-based radiopharmaceuticals, tracing the evolution of FAP-targeted small-molecule inhibitors and highlighting how modifications to linker structures and functional groups critically affect radionuclide delivery to tumor tissue. Their analysis offers a practical framework for the ongoing structural optimization of FAPI tracers intended for both diagnostic imaging and radioligand therapy. Gao et al. addressed the unmet need in pancreatic cancer theranostics by designing DOTA-PEG-PARPi, a polyethylene glycol-modified PARP inhibitor probe labeled with ^68^Ga or ^177^Lu. In preclinical evaluation, the PEG modification preserved high binding affinity while substantially improving pharmacokinetic properties and prolonging tumor retention, with SPECT/CT imaging confirming significant radioactive accumulation in PSN-1 xenografts. Radiation dosimetry using OLINDA/EXM was also performed to guide future clinical dose optimization. Song et al. introduced a synthetic lethality approach for treating pheochromocytomas and paragangliomas. Working with PC12-derived cell lines engineered to overexpress the norepinephrine transporter (NET), they showed that ^131^I-MIBG combined with the PARP inhibitor fluzoparib produced marked synergistic effects, including enhanced G2/M cell cycle arrest and increased apoptosis, particularly in settings where either agent alone was insufficient. Notably, cells with suppressed SDHB expression displayed heightened sensitivity to PARP inhibition, suggesting that genetic background may further modulate therapeutic response. In a methodological contribution, Wang et al. used ^11^C-labeled clozapine-N-oxide (CNO) and clozapine (CLZ) for PET imaging and autoradiography in DREADD mouse models. Their data revealed that brain concentrations of CLZ were approximately 40-fold higher than those of CNO following ^11^C-CNO injection, and that low-dose CLZ administration (0.1 mg/kg) was sufficient to induce robust neuronal silencing in hM4D transgenic mice. These findings establish that CLZ, not CNO, is the principal mediator of both imaging signals and pharmacological effects in murine DREADD systems, providing a critical methodological reference for future DREADD agonist development.

## Molecular imaging for clinical decision-making

Functional molecular imaging is increasingly central to tumor restaging and treatment selection. Wang et al. evaluated ^18^F-AlF-NOTATATE PET/CT for restaging 159 high-risk neuroblastoma patients after chemotherapy. In both lesion-based and patient-based analyses, this somatostatin receptor-targeted tracer demonstrated significantly higher sensitivity, specificity, and accuracy compared with conventional anatomical imaging for detecting residual disease, with a true-positive rate of 88.5% among 634 identified lesions. Building on this platform, Liu et al. prospectively assessed whether quantitative ^18^F-AlF-NOTATATE PET/CT parameters could predict response to ^177^Lu-DOTATATE peptide receptor radionuclide therapy (PRRT) in 22 children with recurrent or refractory neuroblastoma. They found that the ratio of interim-to-baseline lesion SUVmax, with an optimal cutoff of 1.25, achieved the highest predictive accuracy (AUC 0.796), enabling clinicians to identify patients most likely to benefit from PRRT. Extending PET-based metabolic phenotyping to other solid tumors, Zhang et al. analyzed 105 gastric cancer patients and observed that metabolic tumor volume (MTV) and total lesion glycolysis (TLG) were significantly lower in HER2-positive cases, with MTV cutoff of 20.3 cm³ yielding 90.2% accuracy for predicting HER2 positivity. Subgroup analysis further confirmed a significant negative correlation between MTV and HER2 status in gastric adenocarcinoma and Lauren classification subtypes. In advanced non-small cell lung cancer, Luo et al. retrospectively studied 115 patients receiving PD-1/PD-L1 immune checkpoint inhibitors and identified whole-body MTV (MTVwb) as an independent prognostic factor for disease clinical benefit, progression-free survival, and overall survival. They also noted a positive correlation between tumor glycolytic activity and PD-L1 expression in the combination immunotherapy subgroup. Together, these studies underscore the value of PET/CT-derived metabolic parameters as non-invasive biomarkers for characterizing tumor biology and guiding therapeutic decisions across diverse cancer types.

## Conclusion

The studies assembled in this Research Topic encompass drug resistance mechanisms, novel radiopharmaceutical design, and clinical molecular imaging, reflecting the multifaceted progress of nuclear medicine from bench to bedside. By validating new therapeutic targets, optimizing diagnostic and therapeutic probes, and demonstrating the clinical utility of metabolic imaging biomarkers, this collection of work advances the field toward the goal of truly integrated precision theranostics. Looking ahead, continued interdisciplinary collaboration between radiochemists, oncologists, and imaging scientists will be essential to translate these promising findings into routine clinical practice and ultimately improve patient outcomes.

